# Tuning MXene Properties through Cu Intercalation: Coupled Guest/Host Redox and Pseudocapacitance

**DOI:** 10.1021/acsnano.3c12989

**Published:** 2024-03-21

**Authors:** Shianlin Wee, Xiliang Lian, Evgeniya Vorobyeva, Akhil Tayal, Vladimir Roddatis, Fabio La Mattina, Dario Gomez Vazquez, Netanel Shpigel, Mathieu Salanne, Maria R. Lukatskaya

**Affiliations:** † Electrochemical Energy Systems Laboratory, Department of Mechanical and Process Engineering, ETH Zurich, 8092 Zurich, Switzerland; ‡ Physicochimie des Électrolytes et Nanosystèmes Interfaciaux, PHENIX, Sorbonne Université, CNRS, F-75005 Paris, France; § Deutsches Elektronen-Synchrotron DESY, Notkestrasse 85, Hamburg D-22607, Germany; ∥ Helmholtz Centre Potsdam, GFZ German Research Centre for Geosciences, 14473 Potsdam, Germany; ⊥ Empa - Swiss Federal Laboratories for Materials Science and Technology, 8600 Dübendorf, Switzerland; # Department of Chemical Science, Ariel University, Ariel 40700, Israel; ○ Institut Universitaire de France (IUF), 75231 Paris, France

**Keywords:** 2D materials, *in situ* X-ray absorption spectroscopy, *ab initio* molecular dynamics, MXenes, pseudocapacitance, transition metal intercalation, charge storage mechanism

## Abstract

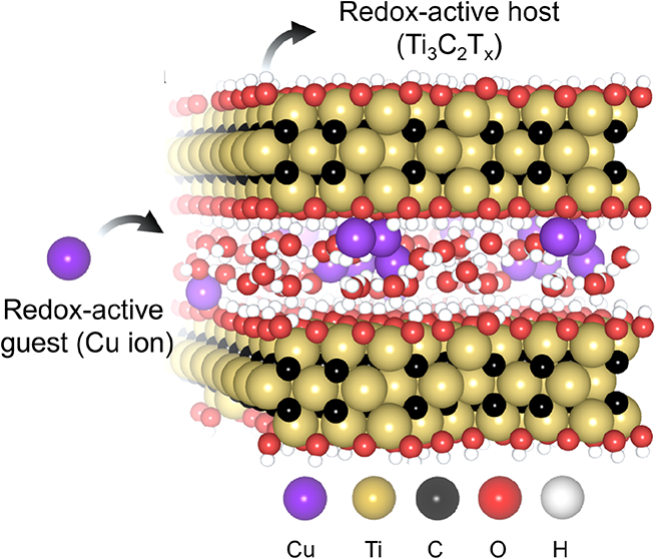

MXenes are 2D transition metal carbides, nitrides, and/or carbonitrides that can be intercalated with cations through chemical or electrochemical pathways. While the insertion of alkali and alkaline earth cations into Ti_3_C_2_T_*x*_ MXenes is well studied, understanding of the intercalation of redox-active transition metal ions into MXenes and its impact on their electronic and electrochemical properties is lacking. In this work, we investigate the intercalation of Cu ions into Ti_3_C_2_T_*x*_ MXene and its effect on its electronic and electrochemical properties. Using X-ray absorption spectroscopy (XAS) and *ab initio* molecular dynamics (AIMD), we observe an unusual phenomenon whereby Cu^2+^ ions undergo partial reduction upon intercalation from the solution into the MXene. Furthermore, using *in situ* XAS, we reveal changes in the oxidation states of intercalated Cu ions and Ti atoms during charging. We show that the pseudocapacitive response of Cu-MXene originates from the redox of both the Cu intercalant and Ti_3_C_2_T_*x*_ host. Despite highly reducing potentials, Cu ions inside the MXene show an excellent stability against full reduction upon charging. Our findings demonstrate how electronic coupling between Cu ions and Ti_3_C_2_T_*x*_ modifies electrochemical and electronic properties of the latter, providing the framework for the rational design and utilization of transition metal intercalants for tuning the properties of MXenes for various electrochemical systems.

## Introduction

MXenes are a family of two-dimensional (2D) transition metal carbonitrides^1^ with a general formula of M_*n*+1_X_*n*_T_*x*_, where M is an early transition metal, *n* = 1–4, X is carbon and/or nitrogen, and T_*x*_ refers to surface termination such as =O, −OH, −Cl, −F, etc.^2−4^ MXenes offer a combination of tunable metallicity^1,5^ and hydrophilicity,^1,5−7^ coupled with attractive redox properties that gave rise to their promise in numerous applications, including fast energy storage,^5,8−15^ electrocatalysis,^16,17^ and biomedical^18−20^ and electromagnetic shielding.^21^

Electronic and electrochemical properties of MXenes can be tailored by changing its chemistry: from the type of transition metals (TMs) within the MX layer to modification of the surface terminations.^1,22−24^ Further, since MXenes are layered and have negatively charged surfaces,^9,25,26^ they can be (electro-)chemically intercalated by various cations and polar molecules^1,9,27−29^ such as monovalent (Li^+^, Na^+^, K^+^, NH_4_^+^),^9,12,30^ multivalent (Mg^2+^, Al^3+^, Sn^4+^),^9,12,31−33^ and organic cations (alkylammonium (TBA)^12,29,34^), offering an additional tuning knob to alter their physicochemical properties.^22,35^ However, very few studies focus on the intercalation of TM cations in MXenes. We suggest that intercalation of TM cations into semimetallic MXenes represents a special scenario, in which redox active intercalants can cause charge redistribution within MXene layers and, thus, alter its electronic properties and electrochemical response, resulting in distinct properties. For example, a previous study showed that immobilization of single Pt atoms on Mo vacancies in Mo_2_TiC_2_T_*x*_ enabled an enhanced hydrogen evolution activity, which was attributed to the changes in the electronic structure of the Pt–Mo_2_TiC_2_T_*x*_ system.^17^ While there have been a few recent reports of TM intercalation in MXenes,^36−38^ there is a lack of understanding regarding the fundamental interactions between these redox-active intercalants and the MXene host.

Herein, we study how Cu intercalation into Ti_3_C_2_T_*x*_ changes its electronic and electrochemical properties. For this, we combine X-ray absorption spectroscopy (XAS), four-point probe measurements, *ab initio* molecular dynamics (AIMD), and density functional theory (DFT) calculations. Specifically, we track the changes in the oxidation states of both intercalated Cu ions and Ti atoms in the MXene using *in situ* XAS under different applied potentials. Our work offers an insight into the complex interactions between intercalated transition metal cations and MXene layers.

## Results and Discussion

### Characterization of Cu–Ti_3_C_2_T_*x*_

**Figure 1:**
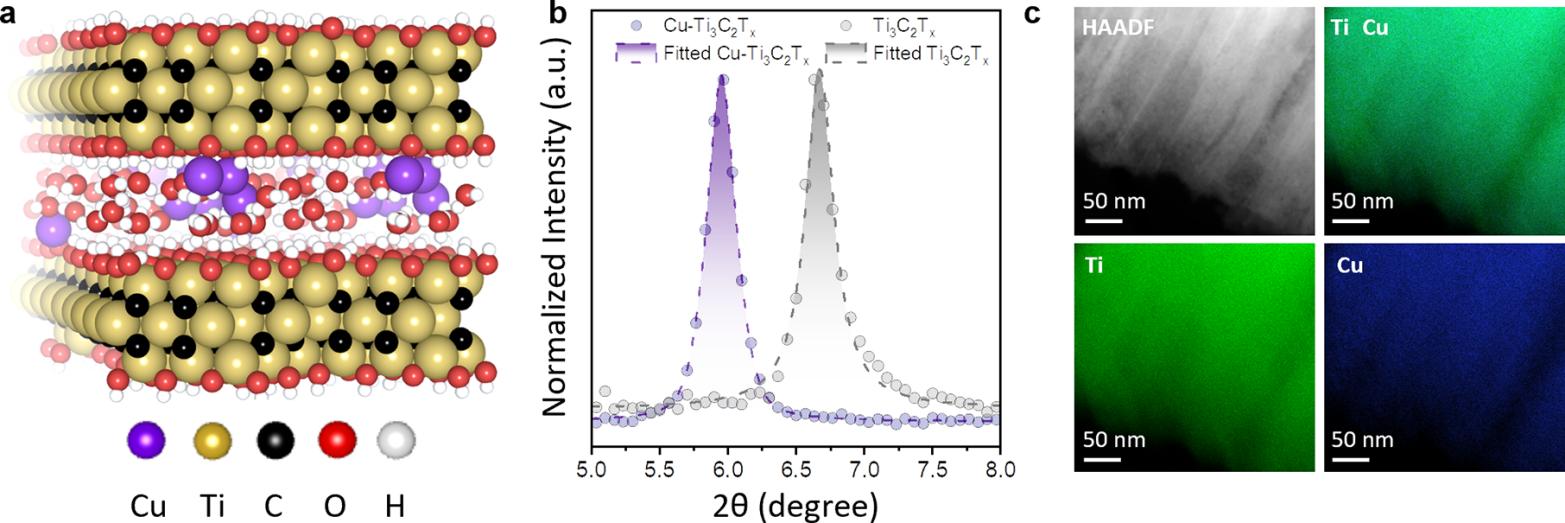
(a) The structure of Cu–Ti_3_C_2_T_*x*_ MXene generated with *ab initio* molecular dynamics (AIMD). (b) X-ray diffraction patterns of the pristine and Cu-intercalated Ti_3_C_2_T_*x*_ showing the (002) peak. (c) STEM images with the corresponding EDX maps of the Ti and Cu elements in Cu-intercalated Ti_3_C_2_T_*x*_.

Cu-intercalated Ti_3_C_2_T_*x*_ MXene was synthesized following the Ghidiu et al. method,^39^ as described in the Materials and Methods section. Intercalation of Cu ions was verified through X-ray diffraction (XRD): the (002) peak shifted to lower angles (2θ = 6.0°) corresponding to a larger d-spacing of 14.8 Å compared to the pristine Ti_3_C_2_T_*x*_ (2θ = 6.7°, d-spacing of 13.2 Å)^40^ (Figures 1b and S1). Scanning transmission electron microscopy (STEM) mapping of chemical elements using energy dispersive X-ray spectroscopy (EDX) reveals a homogeneous distribution of Cu in Ti_3_C_2_T_*x*_ (Figures 1c and S3). Quantification of the intercalated Cu fraction was carried out using inductively coupled plasma-optical emission spectrometry (ICP-OES). It shows that the average Cu content is 0.23 ± 0.007 per Ti_3_C_2_T_*x*_ formula unit (Figure S2), which is close to the values estimated from energy-dispersive X-ray spectroscopy (EDX). Importantly, we confirmed the absence of Cu nanoparticles on the surface of MXene particles using scanning transmission electron microscopy (STEM)-EDX (Figures 1c and S3) and XRD (Figure S1). These results confirm that Cu ions successfully intercalate within the Ti_3_C_2_T_*x*_ structure. Interestingly, the d-spacing in Cu–Ti_3_C_2_T_*x*_ falls in-between values typically attributed for Ti_3_C_2_T_*x*_ with a monolayer and Ti_3_C_2_T_*x*_ with a bilayer of water.^39^ We elucidated this observation further computationally by performing DFT relaxations of structures with various amounts of water, starting from the monolayer case (corresponds to 22 water molecules with our simulation box size) and progressively increasing the number of water molecules. We obtained a good match with experimental d-spacing (14.8 Å) for 30 molecules: that composition corresponds to 1.4 monolayers of water (for our simulation box size). Since the system also contains Cu ions, water molecules cannot “fit” in a single layer and therefore arrange in two layers, as can be seen in the snapshot shown in Figure 1a. Therefore, we denote such structure as a bilayer.

**Figure 2:**
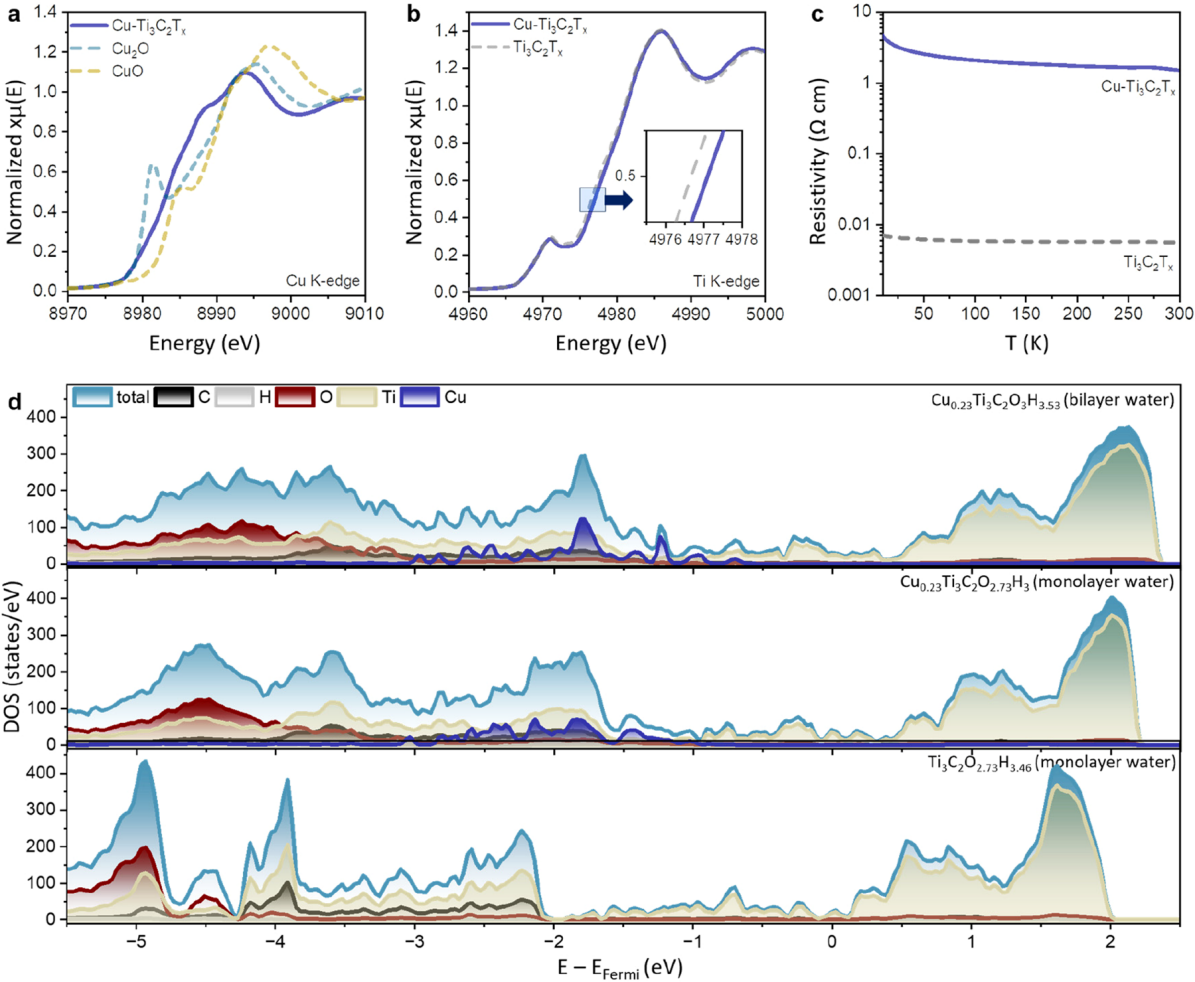
(a) Normalized X-ray absorption spectra of Cu–Ti_3_C_2_T_*x*_, Cu_2_O, and CuO at Cu K-edge. (b) Normalized X-ray absorption spectra of Cu–Ti_3_C_2_T_*x*_ and Ti_3_C_2_T_*x*_ at Ti K-edge. (c) Temperature-dependent resistivity of the pellets from Cu–Ti_3_C_2_T_*x*_ and Ti_3_C_2_T_*x*_ powders. (d) Density of states (DOS) for Cu_0.23_Ti_3_C_2_O_3_H_3.53_ (Cu-MXene with bilayer water, upper panel), Cu_0.23_Ti_3_C_2_O_2.73_H_3_ (Cu-MXene with monolayer water, middle panel), and Ti_3_C_2_O_2.73_H_3.46_ (pristine MXene with monolayer water, lower panel) obtained from DFT calculations.

Next, we investigated the chemical state of intercalated Cu ions using XAS (Figure 2a) and AIMD. XANES spectra of Cu–Ti_3_C_2_T_*x*_ lacks a pre-edge feature that is a known signature of Cu^2+^ (d^9^ electronic configuration, classic 1s → 3d transition) (Figure S5).^41^ Moreover, the energy of the Cu absorption edge in Cu–Ti_3_C_2_T_*x*_ resides between those of Cu_2_O (Cu^1+^) and CuO (Cu^2+^), indicating that the electron density around the Cu ions changes significantly upon intercalation in MXene. To quantitatively estimate the oxidation state of intercalated Cu ions, we compared the XANES spectra of Cu–Ti_3_C_2_T_*x*_, Cu_2_O, and CuO: Figure S6 shows the corresponding edge energy vs Cu oxidation state in known Cu compounds. Using interpolation, an average oxidation state of Cu ions is estimated to be approximately +1.3.

To further rationalize our experimental observation, we performed electronic structure calculations. For this, we employ *ab initio* molecular dynamics (AIMD) because (1) it is difficult to infer the initial positions of Cu atoms in Cu–Ti_3_C_2_T_*x*_ when interlayer water is present; therefore, it is necessary to use MD in order to generate representative configurations, and (2) the complex electronic structure of Cu ions requires accounting for quantum effects. Specifically, we used AIMD to simulate the two cases of Cu^2+^ ions inserted in a water monolayer or bilayer between the Ti_3_C_2_T_*x*_ layers. While the oxidation state is not a direct observable in DFT,^42^ an estimation of the partial charge of Cu atoms obtained through the Bader analysis (Table S1) yields values of +0.21|e| for the bilayer water and +0.42|e| for the water monolayer configurations. Given that the Bader charge of Cu in bulk Cu_2_O is +0.52|e|, our values for Cu–Ti_3_C_2_T_*x*_ show the reduction of Cu^2+^ ions upon their insertion into the MXene layers. Further, in both cases of water monolayer and bilayer, the average Cu–O distance is 1.9 Å, which is in between the typical values for the hydrated Cu(I) (1.8 Å) and Cu(II) (2.0 Å) ions.^43^ Although the Cu atoms display a variety of coordination environments, since Cu coordination number by oxygen atoms is lower than 2 in our simulations (Figure S7), it is likely close to a +1 redox state according to the bulk structure.^43^

Additionally, as our AIMD-generated structures are partially disordered, the total density of states (DOS) calculated via DFT is rather noisy near the Fermi level (Figure 2d) and therefore is difficult to analyze. However, by projecting it over the various elements, we observe in both cases (water monolayer and bilayer) that the Cu states in the valence band are mixed with the Ti and C ones and are located well above the contribution of the water molecules (by approximately 2 eV). In their previous study of aqueous solutions of Cu ions, Blumberger et al. showed that the highest occupied molecular orbital (HOMO) associated with Cu^2+^ ions is almost on top of the HOMOs of the water molecules, while the HOMO of Cu^+^ ion lies 2 eV above.^43^ This further confirms the partial reduction of Cu during intercalation into the MXene.

Concurrently, upon Cu intercalation, the Ti K-edge energy shifts by ∼0.4 eV toward more positive values compared to pristine Ti_3_C_2_T_*x*_ (Figures 2b and S8), showing the partial oxidation of Ti MXene. This is further confirmed by the Bader charge analysis in which the outermost Ti layer in Cu–Ti_3_C_2_T_*x*_ has a higher partial charge compared to the one from pristine Ti_3_C_2_T_*x*_ (Figure S9), consequently leading to the decrease of Ti’s DOS near the Fermi level. Thus, this suggests that the intercalated Cu ions induce charge redistribution within the MXene host and partially accept electron density from the MXene layers.^17,44^ This is further supported by the temperature-dependent electrical conductivity measurements of the pellets. Cu-intercalated MXene shows >2 orders of magnitude increase in resistivity (Figure 2c) compared to the pristine Ti_3_C_2_T_*x*_ that exhibits quasi-metallic behavior.^45^ We attribute the increased resistance of Cu–Ti_3_C_2_T_*x*_ to the decreased density of electrons at the Fermi level induced by the oxidation of Ti upon Cu ion intercalation.

We can therefore confirm that partial oxidation of Ti atoms upon Cu intercalation (observed in XAS, AIMD) leads to the reduction in charge carrier density and thus increases the resistivity in Cu–Ti_3_C_2_T_*x*_. At the same time, Cu DOS does not contribute to the in-plane conductivity that is normally at play in MXene materials.^46^

### Electrochemical Response of Cu–Ti_3_C_2_T_*x*_

**Figure 3:**
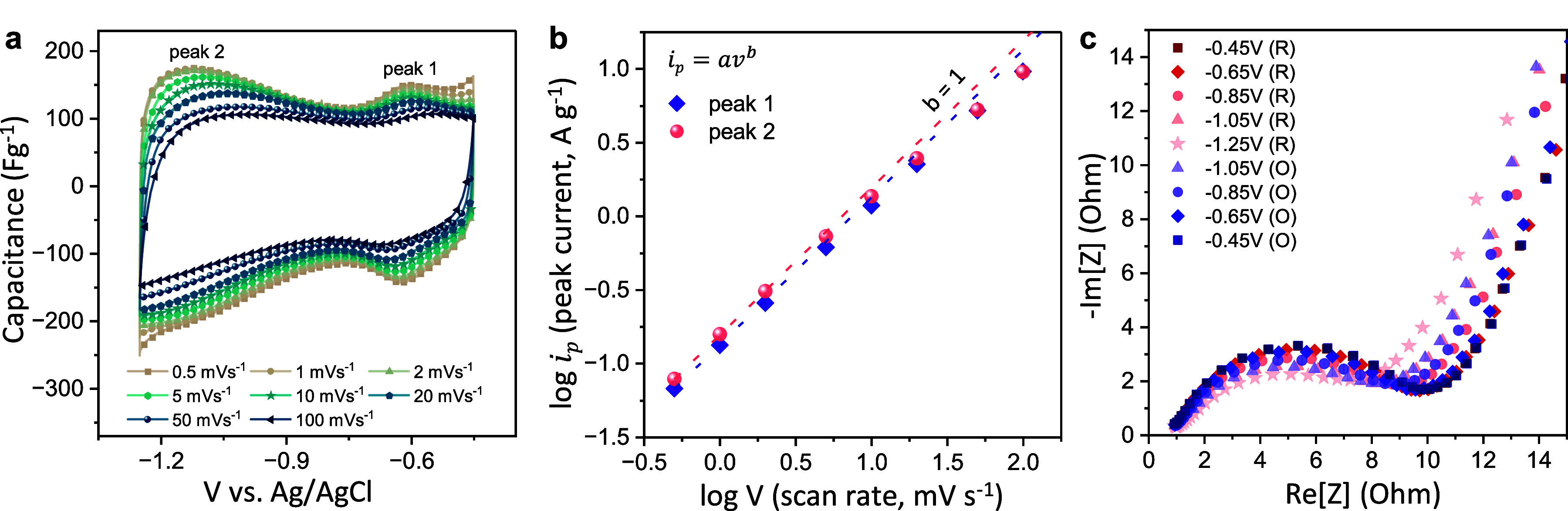
(a) Normalized cyclic voltammetry profiles of Cu–Ti_3_C_2_T_*x*_ at scan rates from 0.5 to 100 mV s^–1^. (b) The logarithm of peak current density versus logarithm of scan rate that shows a *b* coefficient of 1. The dashed lines refer to slope *b* = 1. (c) Electrochemical impedance spectra (EIS) of Cu–Ti_3_C_2_T_*x*_ collected at different applied potentials.

**Figure 4:**
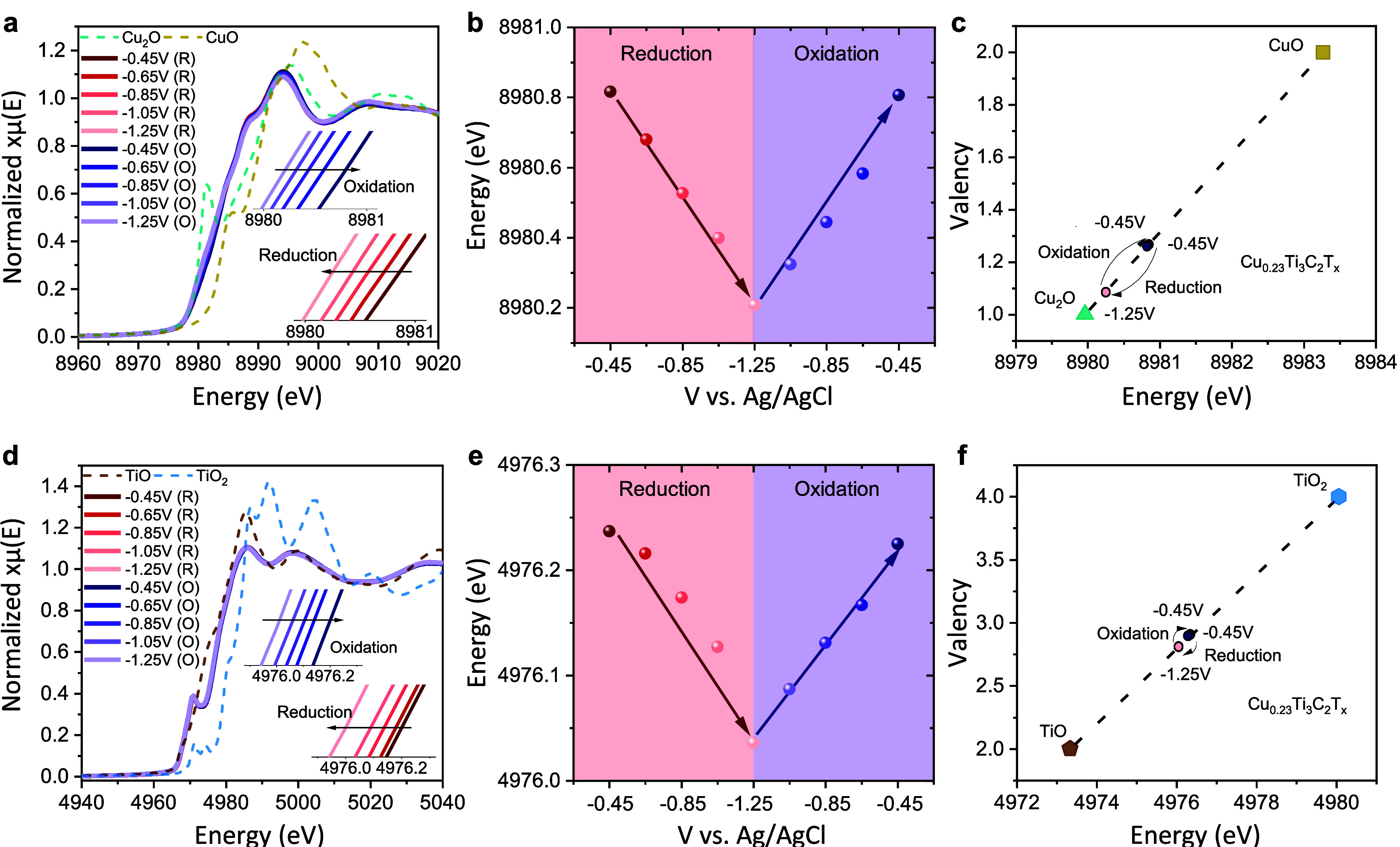
Electrochemical XAS data of Cu–Ti_3_C_2_T_*x*_: (a) Cu K-edge XANES spectra at different applied potentials and (b) variation of Cu edge energy vs potential during negative and positive potential sweep. (c) Average Cu oxidation states at various potentials; Cu K-edge energies of Cu_2_O (+1) and CuO (+2) are added for reference. (d) Ti K-edge XANES spectra of Cu–Ti_3_C_2_T_*x*_ measured at different applied potential. (e) Variation of Ti edge energy with applied potential. (f) Average Ti oxidation state as a function of potential; Ti K-edge energies of TiO (+2) and TiO_2_ (+4) are added for reference.

The electrochemical response of Cu–Ti_3_C_2_T_*x*_ in 1 M NaOH is characterized by the distinct cyclic voltammetry (CV) profile with an additional redox peak at −0.6 V vs. Ag/AgCl was compared to pristine Ti_3_C_2_T_*x*_ (Figures 3a and S11). To characterize charge storage kinetics, we further analyzed peak current (*i*_p_) dependence on the scan rate (*v*) for two redox peaks (denoted peak 1 and peak 2 in Figure 3a) assuming a power-law relationship between the *i*_p_ and *v* as expressed by the equation: *i*_p_ = *av*^*b*^, where *a* and *b* are variables. When plotting the log(*i*_p_) vs log(*v*), it yields a straight line with a slope of *b* ∼ 1 for both peaks in the studied scan rate range (see Figure 3b). The fact that *b* = 1 indicates capacitive charge storage kinetics for Cu–Ti_3_C_2_T_*x*_ samples that is not diffusion-limited (in which case, *b* = 0.5). Next, to examine the redox behavior of intercalated Cu ions in Cu–Ti_3_C_2_T_*x*_, we performed *in situ* XAS of the Cu K-edges at different applied potentials, ranging from −0.45 to −1.25 V vs Ag/AgCl. In the XANES region, a highly reversible shift of the Cu K-edge energy toward lower energies was observed during the cathodic scan, corresponding to a partial reduction of Cu ions (Figure 4a,b). Concurrently, the overall electrode resistance decreases, as observed from the electrochemical impedance spectra (EIS) collected at different reducing potentials (Figure 3c). We estimate that, on average, each Cu ion gains ∼0.2 e^–^ during charging (Figure 4c). It is also important to note that Cu ions in Cu–Ti_3_C_2_T_*x*_ do not undergo full reduction to the Cu^0^ state, even though applied potentials (−1.25 V) are significantly more reducing than the standard reduction potentials of Cu^0^/Cu^2+^ (−0.44 V versus Ag/AgCl)^47^ or Cu^0^/Cu^+^ (−0.58 V vs Ag/AgCl).^47^

Next, we examined the changes in the Ti K-edge energies as a function of the potential. Similar to the trend observed for the Cu K-edge, the Ti XANES spectra also show reversible shifts toward lower energy (decrease in Ti oxidation state) during the negative scan (Figure 4d,e). By correlating edge energy shifts to the Ti valency, we estimate that each Ti_3_C_2_T_*x*_ formula unit gains ∼0.18 e^–^ during charging over the potential window of 0.8 V (Figure 4f). Hence, according to XAS, each Cu_0.23_Ti_3_C_2_T_*x*_ unit gains 0.22 e^–^ during charging, which can be calculated as the redox capacitance of 125 F g^–1^. This value is quite close to the capacitance of 145 F g^–1^ that we obtain directly from the electrochemical measurements in a Swagelok cell. Therefore, we can infer that both Ti and intercalated Cu ions coparticipate in charge storage and make a dominant contribution to the overall capacitance.

Finally, the fact that Ti changes its oxidation state linearly with potential when Cu–Ti_3_C_2_T_*x*_ is cycled in the alkaline electrolytes (Figure 4e) further indicates the pseudocapacitive charge storage mechanism of Ti_3_C_2_T_*x*_ (similar to the mechanism revealed for Ti_3_C_2_T_*x*_ in acidic electrolytes before^48^). Importantly, this observation also does not support the hypothesis that the smaller capacitance of pure Ti_3_C_2_T_*x*_ previously observed in alkaline and neutral electrolytes stems predominantly from the electrical double-layer contribution.^25,49^

## Conclusions

We explored the intercalation of Cu cations into MXene and found several interesting aspects of this process. First, Cu^2+^ ions undergo partial reduction upon intercalation from the solution into the MXene host. This causes a charge redistribution within the MXene layers, leading to a change in its electronic properties and electrochemical response. Second, we reveal that both intercalated Cu ions and Ti inside MXene undergo a reversible redox reaction and participate in charge storage. Third, our experiments suggest that Ti_3_C_2_T_*x*_ and Cu–Ti_3_C_2_T_*x*_ exhibit predominantly pseudocapacitive behavior in alkaline medium with a much smaller electrical double-layer contribution than assumed before. Ultimately, our work demonstrates the feasibility of using transition metal intercalants to tune the properties of MXenes for various electrochemical applications.

## Materials and Methods

### Synthesis of Ti_3_C_2_T_*x*_

Cu-intercalated Ti_3_C_2_T_*x*_ and Ti_3_c_2_T_*x*_ MXene were synthesized following the Ghidiu et al. procedure.^39,40^ Specifically, 1 g of Ti_3_AlC_2_ powder (Carbon-Ukraine, <44 μm particle size) was gradually added to a 10 mL solution of 10 wt % hydrofluoric acid (HF, Sigma-Aldrich, 48 wt %) and ∼1.1 g of LiCl (with a molar ratio 5:1 to Ti_3_AlC_2_). Then, the mixture was stirred at 300 rpm for 24 h at room temperature. This step leads to the simultaneous selective removal of the Al layer from Ti_3_AlC_2_ and the intercalation of Li^+^ ions in between Ti_3_C_2_T_*x*_ layers. Next, the wet sediment was divided into two batches and washed 3 times with 6 M HCl (hydrochloric acid, Sigma-Aldrich, 37%) with a ratio of 0.5 g of MXene to 40 mL of HCl solution. After each washing, centrifugation at 3500 rpm for 5 min was performed. This yielded the Ti_3_C_2_T_*x*_ MXene in which intercalated Li^+^ ions were exchanged by H_3_O^+^. Finally, the sediment was washed with 40 mL of MiliQ water at least 4 times until pH 5 was reached. For the control sample, non-intercalated “pristine” Ti_3_C_2_T_*x*_, the wet sediment was collected directly after DI washing and dried and used as is for the experiments.

### Preparation of Cu-Intercalated Ti_3_C_2_T_*x*_

For the intercalation of Cu ions, 40 mL of 0.1 M copper(II) sulfate pentahydrate (CuSO_4_·5H_2_O, Carl Roth, 99%) was added to 0.5 g of wet Ti_3_C_2_T_*x*_ sediment. The mixture was manually shaken for 2 min and then allowed to rest for 1 h. After centrifugation at 3500 rpm for 5 min, the supernatant was removed and replaced with a fresh 0.1 M CuSO_4_ solution. Then, the mixture was left for another 24 h under continuous stirring at 300 rpm and Ar bubbling at room temperature. Finally, the sediment was washed three times with MiliQ water, collected via vacuum filtration, and dried under vacuum for 24 h.

### Electrode Preparation

The electrodes were prepared following the procedure described in refs (9 and 50). The working electrode was composed of 90 wt % of MXene powder, 5 wt % of polytetrafluoroethylene binder (PTFE, Sigma-Aldrich), and 5 wt % of carbon black (CB, Orion). The counter electrode contained 95 wt % of activated carbon (MTI) and 5 wt % of polytetrafluoroethylene binder (PTFE, Sigma-Aldrich).

### X-ray Diffraction

X-ray diffraction patterns were acquired using a PANalytical Empyrean X-ray Powder Diffractometer with Cu Kα radiation at 40 mA and 40 kV. The powders were scanned in the 2θ range from 4.5° to 60°, with an acquisition step of 0.067° and 700 s per step in reflection geometry.

### Scanning Transmission Electron Microscopy (STEM) and Energy-Dispersive X-ray Spectroscopy (EDX)

STEM images with the corresponding EDX mapping were collected at 200 kV for 5 min on a FEI Talos F200X microscope and equipped with an FEI SuperX detector (Chem S/TEM, ScopeM, ETH Zurich). Samples for scanning transmission electron microscopy were prepared by dusting the grounded powder onto standard nickel mesh lacey carbon support films (EMresolutions, Quantifoil).

### High Resolution STEM Images and Electron Energy Loss Spectroscopy (EELS) Maps

High resolution STEM images and EELS maps were collected on cross-sectional TEM lamellas prepared by focused ion beam (FIB) milling with the use of the HELIOS G4 UC FIB/SEM system. An area of 16 × 2 μm on a sample was selected. The FIB procedures were as follows: first, a 1.7 μm thick Pt layer was deposited to prevent damage to the sample caused by FIB sputtering and Ga-ion implementation. The material from the front and back sides of the region of interest was removed with the FIB operating at an accelerating voltage of 30 kV and a beam current of 9 nA. An “Easy Lift” system was used to lift out cut FIB sections, which were fixed on a half-moon-shaped Ni grid. The front and back surfaces were thinned further at 30 kV and with beam currents of 230–80 pA and at 16 kV with beam currents of 80–50 pA. Final polishing was done at 5 and 2 kV with a beam current of 40 pA. Further, the lamellas were studied using a Cs aberration-corrected at the probe side TEM (TFS Themis Z 80-300, operated at 300 kV, equipped with a Gatan Continuum 1065 Electron Energy Loss Spectrometer). Both Helios G4 UC and Themis Z microscopes are a part of the Potsdam Imaging and Spectral Analysis facility (PISA).

### Scanning Electron Microscopy (SEM)

SEM images with the corresponding EDX analysis were collected using a Hitachi S-4800 microscope at 20 kV for 5 min. A thin layer of MXene powder was spread on the carbon tape. Excess powder was removed with compressed air.

### Inductively Coupled Plasma Optical Emission Spectrometry (ICP-OES) Measurements

ICP-OES measurements were conducted with an Agilent 720 ES instrument. The ICP sample preparation involved the addition of approximately 5–8 mg of Cu–Ti_3_C_2_T_*x*_ powders (stored in drybox) to a 10 mL solution of 20 wt % HNO_3_ (Sigma-Aldrich, 70%) at room temperature for at least 24 h under continuous stirring (100 rpm) until complete dissolution was achieved. After that, the solution was diluted until a concentration of 10 wt % HNO_3_ was reached. Prior to each measurement, instrument calibrations were performed using solutions of CuSO_4_ (Carl Roth, 99%) in a 10 wt % HNO_3_ with concentrations of 0, 10, 50, and 100 ppm of Cu ions where the signals were recorded at the most prominent emission wavelength of 327.395 nm.

### Resistivity Measurements

Utilizing a Quantum Design Physical Property Measurement System (PPMS), temperature-dependent resistivity measurements were conducted over a temperature range of 4 to 300 K. For these measurements, we utilized MXene pellets that were prepared using a standard laboratory press at room temperature with a pressure of 20 MPa. These pellets were cut into rectangular stripes for the measurement. On each pellet, four gold (Au) electrode pads, each measuring between 50 and 100 nm in thickness and 0.1 mm in width, were deposited with a Leica EM ACE200 sputter coater. These pads were 2.5 mm apart. Indium wires were subsequently attached to the Au pads, creating a 4-probe geometry. To ensure optimal electrical contact, a small amount of silver (Ag) paint was additionally applied at the contact points. The resistivity measurements at room temperature were carried out using a four-point probe instrument (Ossila). MXene pellets for these measurements were 0.25 mm-thick and 6 mm in diameter and made using a standard laboratory press at 20 MPa.

### Electrochemical Setup and Measurements

Cyclic voltammetry (CV) and electrochemical impedance spectroscopy (EIS) were performed using Biologic MPG-200 and VSP-300 potentiostats, respectively, with a three-electrode Swagelok cell configuration. The MXene electrode served as the working electrode with a glassy-carbon disk as the current collector (CH instruments), while an activated carbon electrode served as the counter electrode with a Ti rod as the current collector and Ag/AgCl in 1 M KCl (CH instruments) was the reference electrode. 1 M NaOH (Sigma-Aldrich, ≥98%) electrolyte and one layer of polypropylene separator (Celgard 3501) were used to assemble the cell. CV was performed at scan rates of 0.5, 1, 2, 5, 10, 20, 50, and 100 mVs^–1^, respectively. Moreover, EIS was measured at open circuit voltage in frequency range from 100 kHz to 100 mHz, with an amplitude of 10 mV and 6 points per decade.

### X-ray Absorption Spectroscopy (XAS)

XAS measurements were performed at the P64 Advanced X-ray Absorption Spectroscopy beamline in Deutsches Elektronen (DESY) Synchrotron (Hamburg, Germany), using a Si(111) monochromator. Samples were prepared by mixing ∼4 mg of powder sample with ∼60 mg of cellulose (Sigma-Aldrich) and made into a 1 mm-thick pellets that are 13 mm in diameter using a pelletizer die set at 5 ton-force. These pellets were measured in transmission mode for Ti K-edge, while Cu K-edge measurements were performed in fluorescence geometry using a passivated implanted planar silicon (PIPS) detector (positioned at a 90° angle relative to the incident X-ray). For each pellet, data was collected for about 2 min around the Ti K-edge (4.96 keV) and Cu K-edge (8.97 keV); a total of 5 acquisitions for both transmission and fluorescence signals were collected. Energy calibration for each spectrum was performed using Cu (edge at 8.97 keV) and Ti (edge at 4.96 keV) foils. The data was averaged for 5 acquisitions and analyzed using Athena software. The Cu edge energy for each sample was defined as energy at the normalized intensity of 0.27 (this intensity corresponds to the zero of the second derivative^51−53^ and a maximum of the first derivative^54^ for the Cu foil spectrum). The Ti edge energy position was extracted based on the half height of the normalized intensity of spectrum^48,51,54^ which is close to the zero of the second derivative.^51−53^

### *In Situ* X-ray Absorption Spectroscopy (XAS)

XAS was performed using a Si(111) monochromator and PIPS detector at the P64 Advanced X-ray Absorption Spectroscopy beamline at DESY Synchrotron (Hamburg, Germany). Both Ti K-edge (4.96 keV) and Cu K-edge (8.97 keV) were collected in fluorescence mode, where the PIPS detector was placed at 45° relative to the MXene electrode in the *in situ* cell. A three-electrode *in situ* cell (ECC-Opto-Std test cell, EL-cell, Germany) was used: 50 nm Au evaporated on 1 mm-thick poly(ether imide) sheet was utilized as both the cell’s X-ray window and a current collector for the Cu-intercalated Ti_3_C_2_T_*x*_ MXene working electrode (90% MXene, 5% PTFE, 5% CB). Overcapacitive activated carbon served as the counter electrode; leakless Ag/AgCl with filling electrolyte 3.4 M KCl (eDAQ) served as reference electrode, and one layer of polypropylene separator (Celgard 3501) was used during the cell assembly. Prior to XAS measurement, the cell was precycled at 1 mVs^–1^ for 3 CV cycles between −0.45 and −1.25 V vs Ag/AgCl. Next, for the *in situ* XAS data acquisition, linear sweep voltammetry (LSV) followed by about a 15 min potential hold was performed for each potential of interest (−0.45, −0.65, −0.85, −1.05, −1.25 V). The *in situ* XAS data were collected for at least 10 min in total during the potential hold, and at least 3 spectra were collected at each potential.

The data analysis was performed by using Athena software. The Cu and Ti edge positions were defined as specified above. An estimation of the Cu oxidation state for Cu–Ti_3_C_2_T_*x*_ samples was performed by referencing edge energy and Cu valence to those of reference compounds Cu_2_O (Sigma-Aldrich, ≥99.99%) and CuO (Sigma-Aldrich, ≥99.0%) (Figure 4c). The changes in the Ti oxidation state were assessed in a similar manner, using TiO (Alfa Aesar, 99.5%) and TiO_2_ rutile (Sigma-Aldrich, 99.5%) reference compounds (Figure 4f).

The valency changes obtained from the Cu and Ti XANES spectra were further correlated with the experimental capacitance values calculated from cyclic voltammetry. To estimate charge storage contributions from Ti and Cu redox, the following formula  was used, where *C*_g_ [F g^–1^] refers to gravimetric capacitance, *z* corresponds to the number of electrons participating in the electrochemical reaction, *F* [96485 C mol^–1^] is the Faraday’s constant, *M*_w_ [g mol^–1^] is the molar weight, and *V* [V] is the potential window. The electrochemical reaction that occurred is assumed as follows:
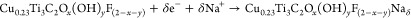
In the case of Cu, the number of electrons participating is equal to 0.19 × 0.23 = 0.04, while for Ti, the number of electrons participating is equal to 0.06 × 3 = 0.18. Assuming a molecular weight of 215.5 g mol^–1^ for Cu_0.23_Ti_3_C_2_T_*x*_, capacitance of 125 F g^–1^ was estimated as a result of Cu and Ti redox contributions. This matches very well with the experimental capacitance of 145 F g^–1^ (calculated capacitance from the CV charge).

### Density Functional Theory (DFT) Calculation and Molecular Dynamics (MD) Simulation

In this work, we used AIMD calculations (procedure described below) since it allows us to overcome the shortcomings of (1) classical MD, which does not account for the redox reactions in a system, and (2) static density functional theory (DFT) that is not well adapted to complex disordered systems such as the aqueous interlayer.

All our calculations were performed within the density functional theory framework as implemented in the Vienna *ab initio* simulation package (VASP).^55,56^ The wave functions are expanded in plane wave basis sets and the projector augmented wave (PAW) method. The exchange-correlation energy is approximated with the generalized gradient approximation as formulated by Perdew, Burke, and Ernzerhof (PBE).^57^ The initial structure with one single layer of water molecules has been reported in ref (58) and was used as a starting point. To lower the computational cost, we reduced the *a* and *b* dimensions, resulting in a structure that contains 90 Ti atoms, 60 C atoms, 82 O atoms, and 104 H atoms. This structure contains 22 H_2_O molecules in between Ti_3_C_2_T_*x*_ layers. Experimentally, each Ti_3_C_2_T_*x*_ unit corresponds to 0.23 ± 0.007 Cu; therefore, we need to insert 7 Cu atoms into the water layer with 14 H atoms randomly removed concurrently.

First, we created the Cu–Ti_3_C_2_T_*x*_ structure starting from the original single-layer water Ti_3_C_2_T_*x*_ structure by adding 7 Cu^2+^ ions to the water layer one by one, followed by geometry optimization and AIMD to stabilize the structure. The final structure contains 90 Ti atoms, 60 C atoms, 82 O atoms, and 90 H atoms, based on the performed AIMD calculation for 35 ps. However, this structure displayed a too low *c* lattice parameter (after relaxation) since the d-spacing was 12.9 Å instead of 14.8 Å in the experiments. The *c* dimension was therefore increased by progressively increasing the number of water molecules. The initial structure of the interlayer (prior to relaxation) was generated by classical molecular dynamics to equilibrate the initial configuration and then inserted into the enlarged MXene layer. The as-prepared structure was relaxed in order to determine the corresponding d-spacing. Good agreement with the experimental value (14.9 Å instead of 14.8 Å) was obtained for a structure that contains 30 H_2_O molecules within the Ti_3_C_2_T_*x*_ layers, corresponding to 90 Ti atoms, 60 C atoms, 90 O atoms, 106 H atoms, and 7 Cu ions. After relaxation, the system adopted a bilayer water structure. Starting from this configuration, we made 10 ps of AIMD production calculations to sample the structure of the system.

For all of the structural optimizations, we set the kinetic energy cutoff to 520 eV and sampled the Brillouin zone with a k-spacing of 0.3 Å^–1^. The electronic convergence threshold is set to 10^–6^ eV, and the forces are converged to 0.05 eV Å^–1^. The trajectories obtained from AIMD were used to calculate the radial distribution functions (RDFs) and the coordination number. We computed the average Bader charges^59^ and the density of states (DOS) based on the final structure after AIMD simulations. The AIMD calculations were performed with an NVT ensemble, where we fixed the number of atoms, volume, and temperature with a time step of 1 fs. The gamma point was used to sample the Brillouin zone with a reduced cutoff energy of 450 eV to make the computation more affordable.

In addition, we took Cu_2_O (ICSD code: 143828) and CuO (ICSD code: 133363) from ICSD and computed the Bader charges, respectively, to make comparisons with Cu intercalated Ti_3_C_2_T_*x*_. The initial experimental structures were relaxed first with a cutoff energy of 520 eV and a k-spacing of 0.2 Å^–1^. The forces are converged to 0.001 eV Å^–1^ during geometry, and the energy convergence threshold is set to 10^–7^ eV.

## Supporting Information Available

XRD data for the Ti_3_AlC_2_ MAX phase, pristine Ti_3_C_2_T_*x*_,Cu–Ti_3_C_2_T_*x*_, and Mg–Ti_3_C2T_*x*_; Cu loading obtained from ICP-OES, STEM-EDX, and SEM-EDX; STEM-EDX of Cu–Ti_3_C_2_T_*x*_; STEM-EELS mapping of Cu–Ti_3_C_2_T_*x*_; Cu K-edge XANES of Cu–Ti_3_C_2_T_*x*_; estimation of Cu and Ti oxidation state through XAS data; coordination number of Cu from AIMD calculations; resistivity measurement data for Cu–Ti_3_C_2_T_*x*_, Mg–Ti_3_C_2_T_*x*_, and Ti_3_C_2_T_*x*_; CV data for Cu_0.23_Ti_3_C_2_T_*x*_ and Ti_3_C_2_T_*x*_ MXenes; Bader charge analysis data for Cu_0.23_Ti_3_C_2_O_3_H_3.53_ (Cu-MXene with bilayer water), Cu_0.23_Ti_3_C_2_O_2.73_H_3_ (Cu-MXene with monolayer water), and Ti_3_C_2_O_2.73_H_3.46_ (MXene with monolayer water) (PDF)

## Notes

The preprint version of this manuscript can be found in ChemRxiv.^60^
