# Supplementary material for: Tuning MXene Properties through Cu Intercalation: Coupled Guest/Host Redox and Pseudocapacitance
Source: ACS Nano. 2024 Mar 21;18(14):10124–32. doi: 10.1021/acsnano.3c12989 (PMC11008361; doi:10.1021/acsnano.3c12989)
Supplement: Supplementary file 1 — nn3c12989_si_001.pdf [file nn3c12989_si_001.pdf]

Tuning MXene Properties through Cu Intercalation: Coupled Guest/Host Redox and Pseudocapacitance

Shianlin Wee[1], Xiliang Lian[2†], Evgeniya Vorobyeva[1†], Akhil Tayal[3], Vladimir Roddatis[4], Fabio La Mattina[5], Dario Gomez Vazquez[1], Netanel Shpigel[6], Mathieu Salanne[2,7], Maria R. Lukatskaya[1*]

[1] Electrochemical Energy Systems Laboratory, Department of Mechanical and Process Engineering, ETH Zurich, 8092 Zurich, Switzerland.
[2] Physicochimie des Électrolytes et Nanosystèmes Interfaciaux, PHENIX, Sorbonne Université, CNRS, F-75005 Paris, France.
[3] Deutsches Elektronen-Synchrotron DESY, Notkestrasse 85, Hamburg D-22607, Germany.
[4] Helmholtz Centre Potsdam, GFZ German Research Centre for Geosciences, 14473 Potsdam, Germany.
[5] Empa - Swiss Federal Laboratories for Materials Science and Technology, 8600 Dübendorf, Switzerland.
[6] Department of Chemical Science, Ariel University, Ariel 40700, Israel.
[7] Institut Universitaire de France (IUF), 75231 Paris, France.

[†] Equal contribution * Corresponding author email: mlukatskaya@ethz.ch

TABLE OF CONTENT

FIGURE S1 2

FIGURE S2 2

FIGURE S3 3

SUPPLEMENTARY DISCUSSION ON HIGH RESOLUTION STEM IMAGING AND EELS MAPPING OF CU-$TI_3C_2T_X$ 4

FIGURE S4 4

FIGURE S5 5

FIGURE S6 5

FIGURE S7 6

FIGURE S8 6

FIGURE S9 7

SUPPLEMENTARY DISCUSSION ON RESISTIVITY OF INTERCALATED $TI_3C_2T_X$ 8

FIGURE S10 8

FIGURE S11 9

TABLE S1 10

SUPPLEMENTARY REFERENCES 11

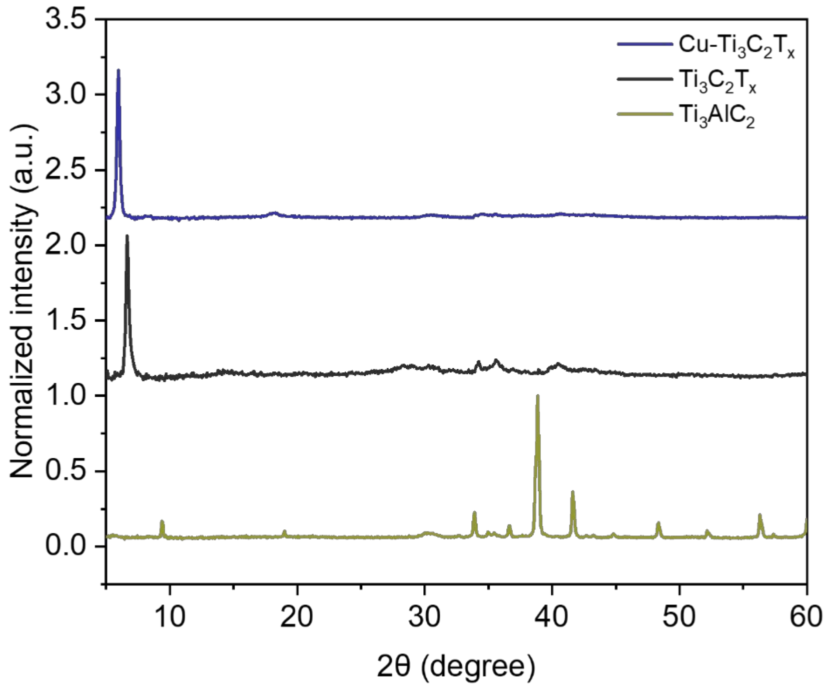

Figure S1. XRD patterns of Ti_3AlC_2 MAX phase, pristine $Ti_3C_2T_x$ and Cu-$Ti_3C_2T_x$ powders.

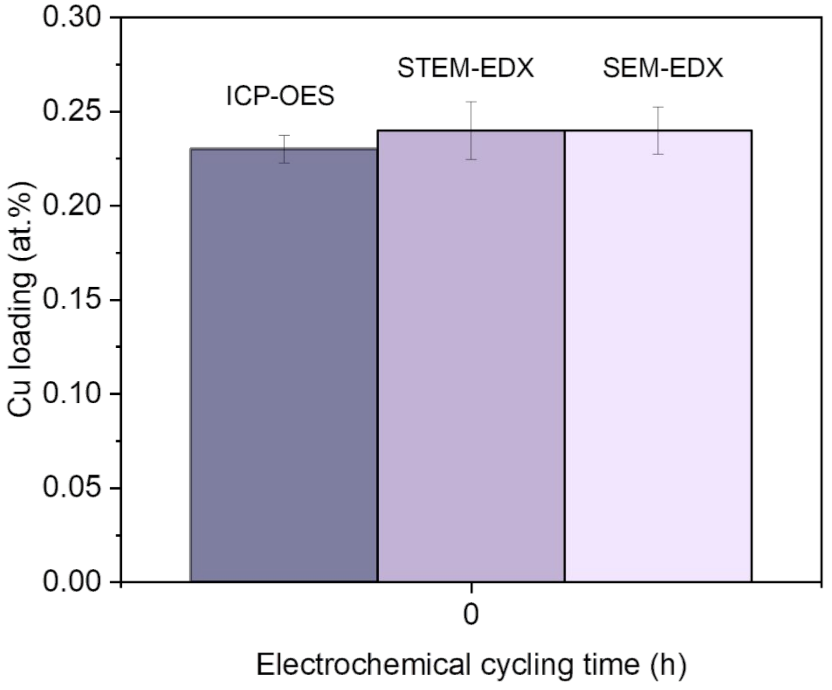

Figure S2. The loading of Cu was quantified through ICP-OES, STEM-EDX and SEM-EDX.

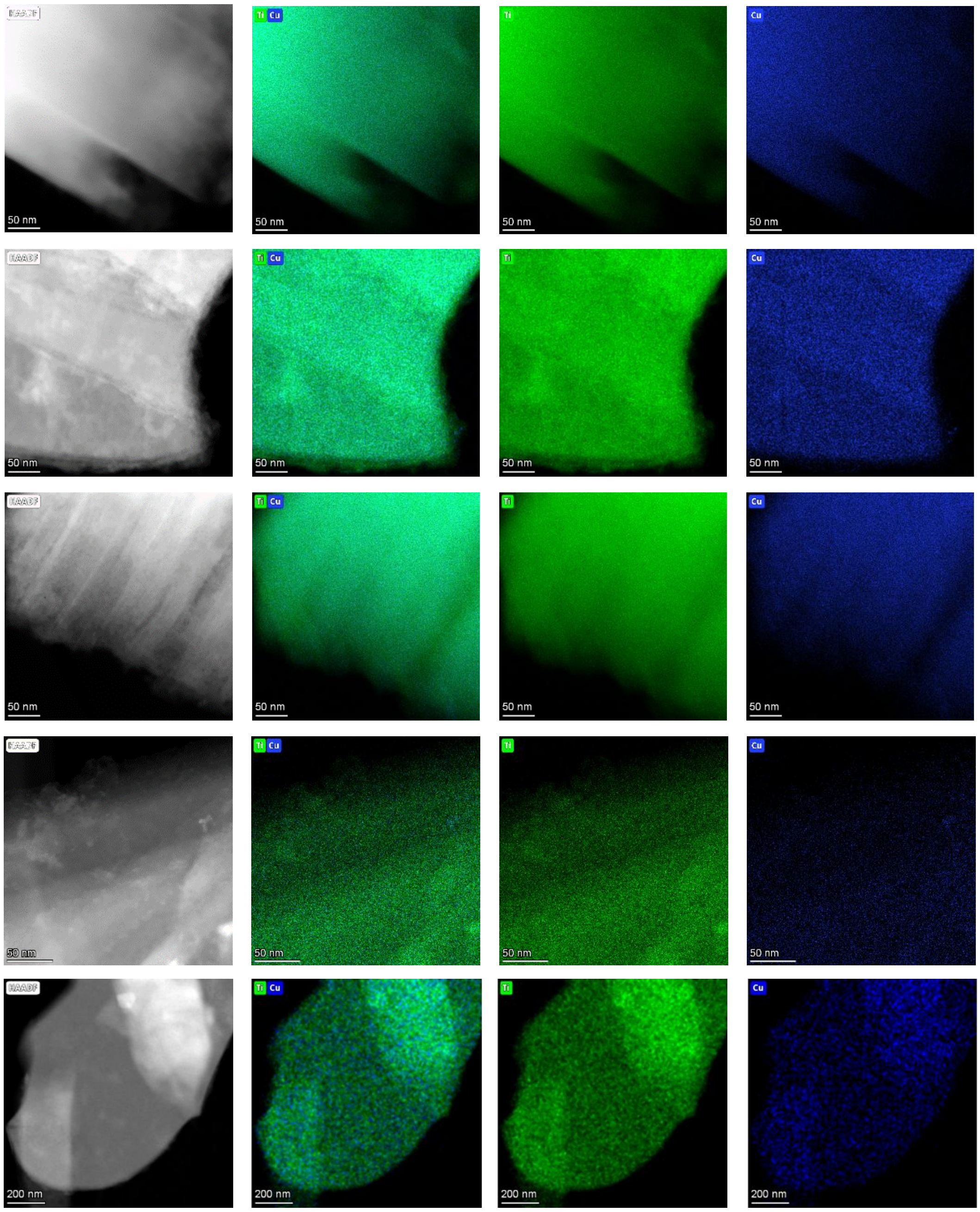

Figure S3. STEM-EDX of Cu-$Ti_3C_2T_x$ multilayer particles.

Supplementary discussion on high resolution STEM imaging and EELS mapping of Cu-$Ti_3C_2T_x$

To be able to visualize MXene layers at atomic resolution, ultrathin (20-40nm) lamellas have to be prepared using focused ion beam (FIB). We prepared such lamellas using FIB for further STEM analysis and electron energy loss spectroscopy mapping (EELS) (**Figure S4**).

Although we were able to atomically resolve MXene layers, the d-spacings calculated from STEM images were mostly in the range from 12.5 to 13.3 Å, which is notably lower than 14.8 Å obtained from XRD (**Figure S4a**). This indicates that the preparation procedure caused damage in the sample, in particular affecting the intercalated water molecules and ions, and thus resulting in reduced interlayer spacing. In addition, structural rearrangement and chemical modification could take place under electron irradiation[S1, S2]. Samples containing water are notoriously difficult to prepare and image due to high beam sensitivity[S3] and require special preparation procedure such as cryo-FIB and use of cryo-EM for imaging to mitigate such damage[S4]. Similarly, EELS mapping also shows the damage: while intercalated Cu ions can be seen in between some of MXene layers (**Figure S4b**), Cu amounts from EELS was lower (approx. 2.6 wt.%) than from ICP or STEM EDX (approx. 6.7 wt.%).

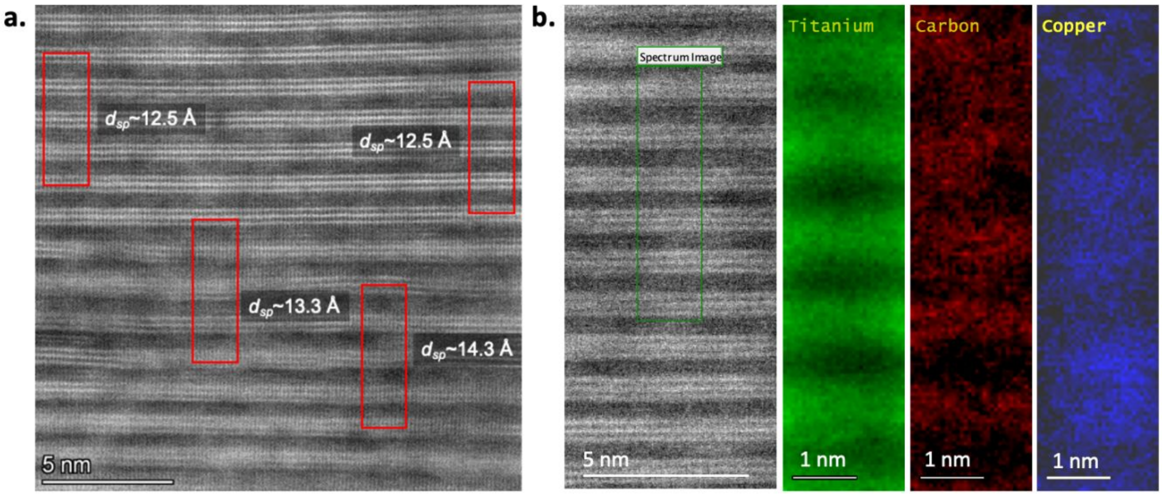

Figure S4. Scanning transmission electron microscopy (STEM) images and electron energy loss spectroscopy (EELS) mapping of Cu-$Ti_3C_2T_x$ multilayer particles. **a.** The d-spacings (d_{sp}) at different locations. **b.** The EELS elemental maps of Ti, C and Cu

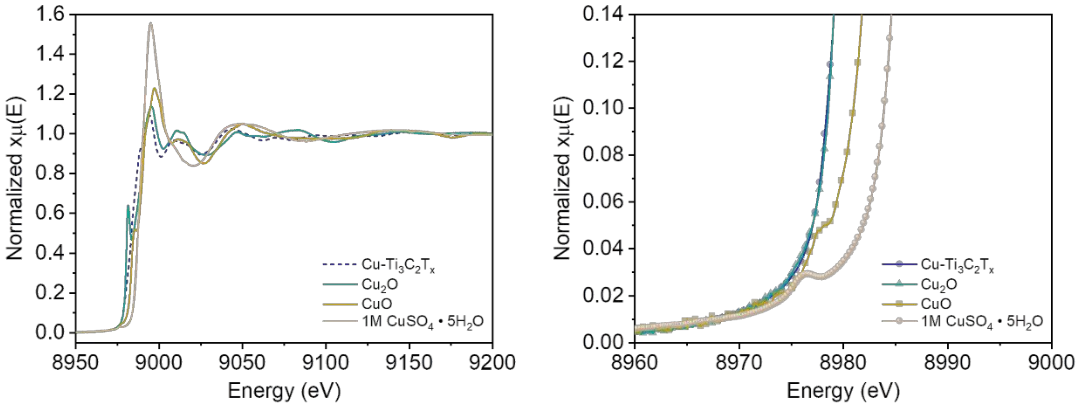

Figure S5. The absence of Cu pre-edge in Cu-$Ti_3C_2T_x$ XAS spectrum.

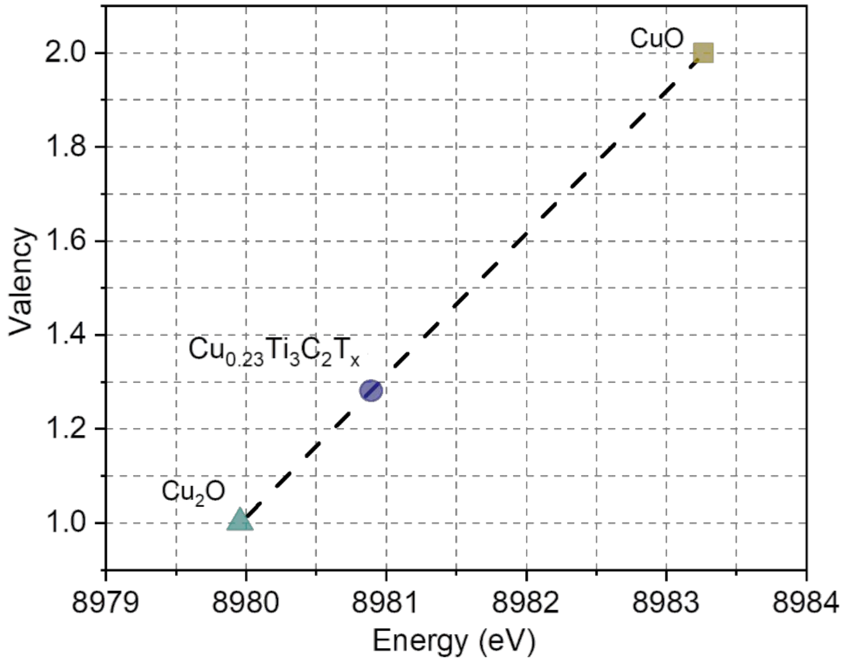

Figure S6. An approximation of Cu oxidation state of Cu-$Ti_3C_2T_x$ through the linear interpolation between Cu_2O (+1) and CuO (+2) energy references.

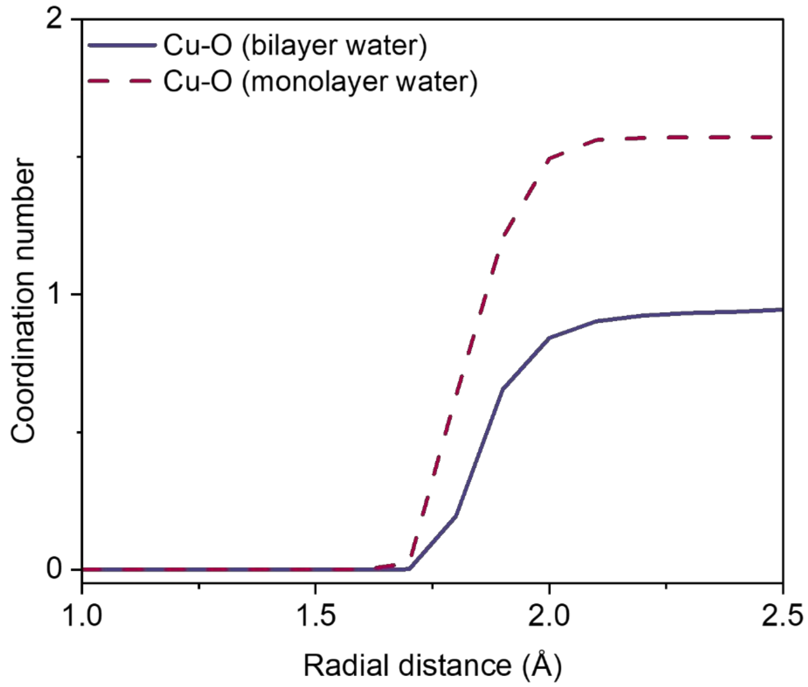

Figure S7. The Cu coordination number by number of oxygens in Cu-intercalated MXene in both bilayer and monolayer water configurations.

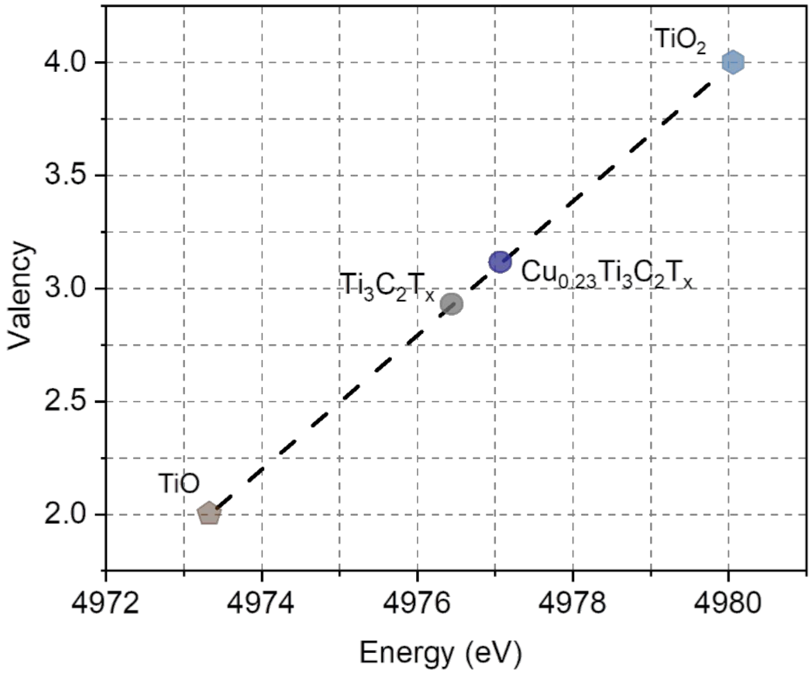

Figure S8. An approximation of Ti oxidation state $Ti_3C_2T_x$ through the linear interpolation between TiO (+2) and TiO_2 (+4) energy references.

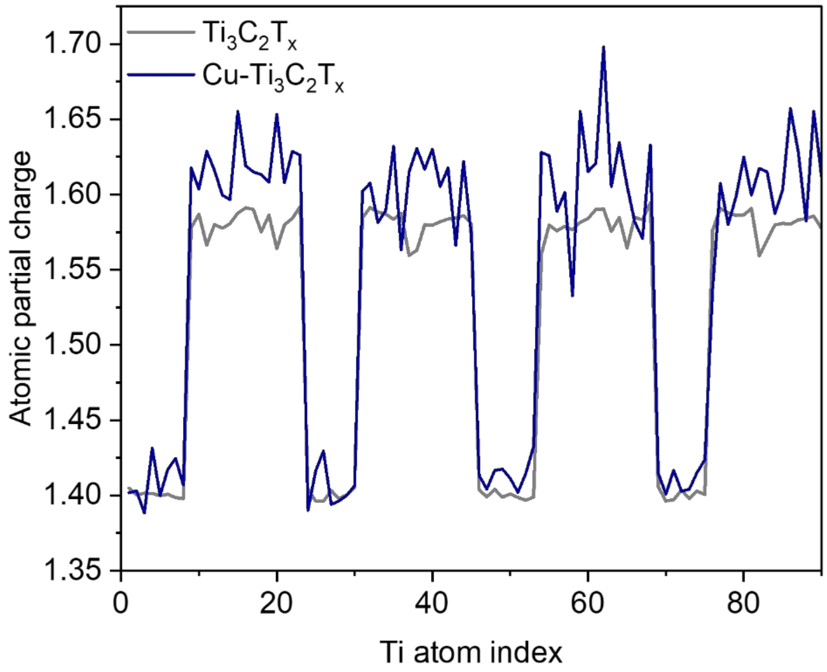

Figure S9. Ti atoms partial charge. The blue line corresponds to Ti partial charges of MXene after Cu intercalation. The grey line corresponds to Ti partial charges of pristine MXene. The Ti atoms with partial charge +1.4 can be ascribed to inner Ti layer and the ones with partial charge around +1.5 to 1.6 are those outermost Ti layer.

Supplementary discussion on resistivity of intercalated $Ti_3C_2T_x$

We observed a resistance increase of two orders of magnitude in Cu-intercalated MXene when compared to pristine MXene (see **Figure 2c**). This led us to explore the impact of increased d-spacing resulting from intercalation versus the intercalant nature. For this purpose, we synthesized a Mg^{2+} intercalated sample (Mg-$Ti_3C_2T_x$) which has a d-spacing comparable to Cu-$Ti_3C_2T_x$ (refer to **Figure S10a**). Mg^{2+} intercalant is redox silent in contrast to copper. We then measured its resistivity (refer to **Figure S10b**). The Mg-$Ti_3C_2T_x$ sample showed an increased resistivity compared to pristine MXene; however, this resistivity was at least an order of magnitude lower than that of Cu-$Ti_3C_2T_x$ despite both samples having comparable interlayer distance. This finding supports our hypothesis that the transport properties of intercalated MXene are influenced not only by the increased interlayer spacing, but also by the nature of intercalant and the charge redistribution upon TM intercalation.

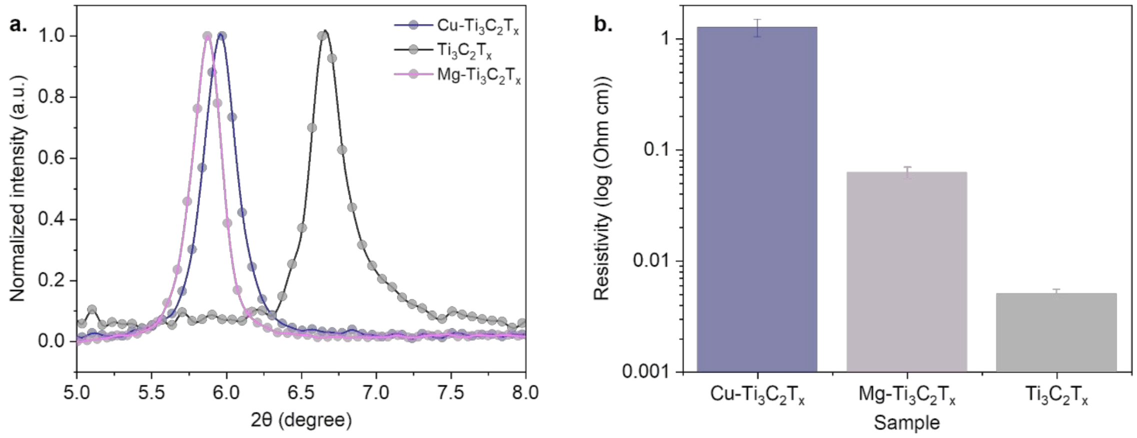

Figure S10. a. XRD patterns of Cu- and Mg-intercalated $Ti_3C_2T_x$, and pristine $Ti_3C_2T_x$. **b.** Resistivity measurements of Cu-$Ti_3C_2T_x$, Mg-$Ti_3C_2T_x$, $Ti_3C_2T_x$ samples using Ossila four-point probe at room temperature.

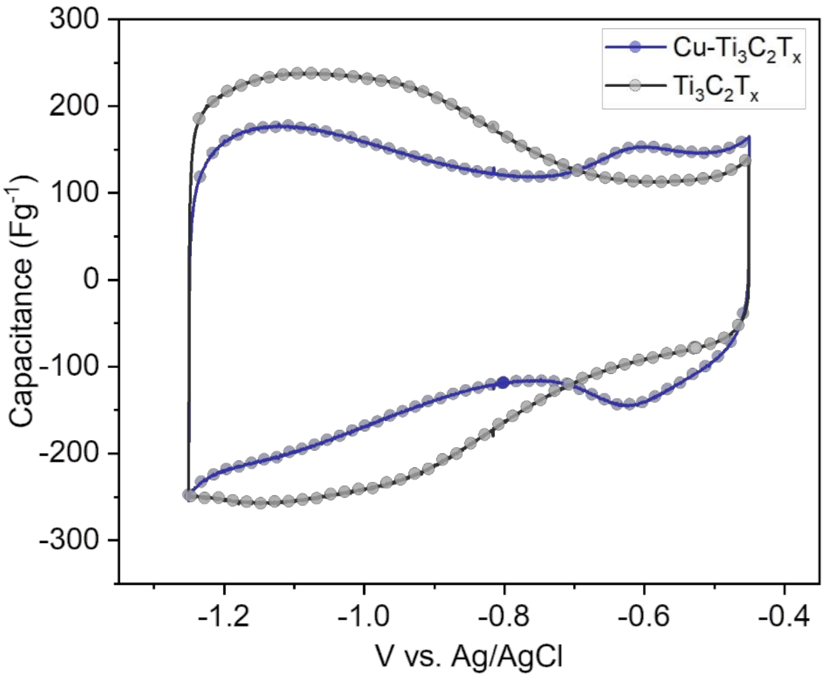

Figure S11. The cyclic voltammetry of $Cu_{0.23}Ti_3C_2T_x$ and $Ti_3C_2T_x$ MXenes at 0.5 mVs^{-1}in 1M NaOH.

Table S1. The Bader charge analysis of both $Cu_{0.23}Ti_3C_2O_3H_{3.53}$ (Cu-MXene with bilayer water), $Cu_{0.23}Ti_3C_2O_{2.73}H_3$ (Cu-MXene with monolayer water), and $Ti_3C_2O_{2.73}H_{3.46}$ (MXene with monolayer water).

Sample	Atom	Bader Charge |e|
$Cu_{0.23}Ti_3C_2O_3H_{3.53}$ (Cu-MXene with bilayer water)	Cu	+0.2119
	Ti	+1.5438
	C	-1.5689
	O	-1.2180
	H	+0.5974
$Cu_{0.23}Ti_3C_2O_{2.73}H_3$ (Cu-MXene with monolayer water)	Cu	+0.4211
	Ti	+1.5421
	C	-1.5743
	O	-1.2193
	H	+0.5857
$Ti_3C_2O_{2.73}H_{3.46}$ (MXene with monolayer water)	Ti	+1.5209
	C	-1.5902
	O	-1.2491
	H	+0.5861

Supplementary References

[S1] Alnoor, H., Elsukova, A., Palisaitis, J., Persson, I., Tseng, E.N., Lu, J., Hultman, L. and Persson, P.Å., 2021. Exploring MXenes and their MAX phase precursors by electron microscopy. *Materials Today Advances*, *9*, p.100123.

[S2] Mendes, R.G., Ta, H.Q., Yang, X., Li, W., Bachmatiuk, A., Choi, J.H., Gemming, T., Anasori, B., Lijun, L., Fu, L. and Liu, Z., 2020. In Situ N-Doped Graphene and Mo Nanoribbon Formation from $Mo_2Ti_2C_3$ MXene Monolayers. *Small*, *16*(5), p.1907115.

[S3] Greer, H.F. and Zhou, W., 2011. Electron diffraction and HRTEM imaging of beam-sensitive materials. *Crystallography reviews*, *17*(3), pp.163-185.

[S4] Burrows, N.D. and Penn, R.L., 2013. Cryogenic transmission electron microscopy: aqueous suspensions of nanoscale objects. *Microscopy and Microanalysis*, *19*(6), pp.1542-1553.
